# Antiepidermal Growth Factor Receptor Monoclonal Antibodies: Applications in Colorectal Cancer

**DOI:** 10.1155/2012/198197

**Published:** 2012-10-08

**Authors:** Efat Azizi, Adam Kittai, Peter Kozuch

**Affiliations:** ^1^Beth Israel Medical Center, Phillips Ambulatory Care Center, Continuum Cancer Centers of New York, 10 Union Square East, Suite 4C, New York, NY, USA; ^2^Section of Hematology/Oncology, Department of Medicine, Albert Einstein College of Medicine, Bronx, NY, USA

## Abstract

Patients with metastatic colorectal cancer have a poor prognosis and present a challenge to clinicians. The role of the antiepidermal growth factor receptor (EGFR) pathway in tumorogenesis and tumor progression has been well defined. This paper will review the use of anti-EGFR monoclonal antibodies in the treatment of operable, as well as metastatic colorectal cancer both in the setting of KRAS mutation unselected patients and later in KRAS wild-type patients. Active investigations designed to further identify predictive biomarkers that may be potentially druggable are reviewed as well.

## 1. Introduction

Colorectal cancer is the third most commonly diagnosed cancer and the third leading cause of cancer death in both men and women in the US [1]. It is estimated that over 140,000 new cases of colorectal cancer (CRC) were diagnosed in 2011 and approximately 50,000 died of this disease [2]. Until a little more than a decade ago the only drug approved for this disease was fluorouracil (5FU), and the median survival with stage IV disease was 12 months. With the development of drugs such as irinotecan and oxaliplatin, the median survival associated with this disease has increased to over 20 months. The ongoing development of antiepidermal growth factor receptor (EGFR) agents and the identification of predictive markers to identify enriched populations who will benefit from anti-EGFR therapy represent active areas of clinical and translational research. This paper will acquaint readers with the pathophysiology that guided the development of anti-EGFR therapies for colorectal cancer and will synthesize the huge amount of clinical data that supports limiting the use of cetuximab and panitumumab alone or in combination with irinotecan as second- or third-line therapy for metastatic colorectal cancer without mutations of the KRAS gene.

## 2. EGFR

The EGFR is a cell surface 170,000 dalton tyrosine kinase transmembrane receptor and a member of the human epidermal growth factor receptor (HER)-ErbB family of receptor tyrosine kinases [3]. Dysregulation of the EGFR pathway occurs in a variety of ways including genetic mutation, gene amplification, protein overexpression, structural rearrangement, and autocrine ligand production [4].

**Figure 1:**
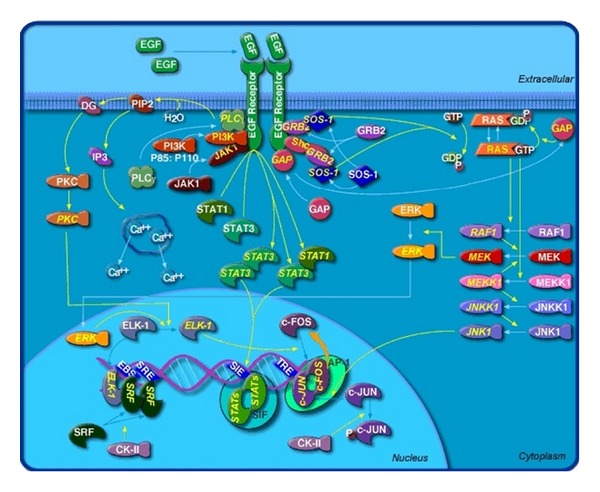
EGFR signaling pathway (reprinted with permission from BioCarta Pathways. All rights reserved).

The ErbB family is composed of 4 transmembrane receptors that interact with each other: EGFR/ErbB1/Her1, ErbB2/Her2/neu, ErbB3/Her3, and ErbB4/Her4 [3–5]. This interaction can result in either homodimerization or heterodimerization. Following dimerization, the intracellular tyrosine kinase portion is phosphorylated leading to downstream activation of complex interacting signaling pathways which include the Ras/Raf/MEK/ERK and the Ras/PI13 K/PTEN//AKT/mTOR pathways [5]. These pathways have been shown to regulate cellular replication, invasion, cellular repair, protection from insult, and induction of apoptosis. As diagrammed in Figure 1, signaling is thought to operate via both vertical and horizontal pathways. As intracellular signaling is found to be a vastly complex network, there is increasing rationale to target more than one signaling pathway or multiple targets within a single pathway in order to effectively regulate cancer. The design of an anticancer therapy employing an inhibitor of EGFR function was hypothesis-driven, based on knowledge available in the early 1980s [6]. EGFR and the Src oncogene product were shown to have the novel enzymatic activity of a tyrosine kinase [6]. Subsequent studies established that EGFR was a cellular oncogene and demonstrated that high levels of EGFR correlated with poorer prognosis in solid tumors [6]. Preclinical studies hypothesized that blockade of the EGFR binding sites with an “antireceptor” monoclonal antibody (mAb) would lead to the inhibition of cell growth, thereby making it an effective anticancer therapy [6].

## 3. EGFR Antagonists

**Figure 2:**
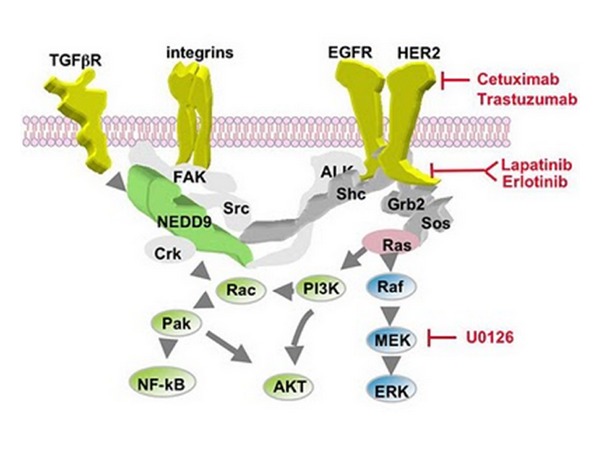
Reprinted with permission from Erica A. Golemis, Ph. D. All rights reserved.

There are two classes of EGFR antagonists currently in clinical use: anti-EGFR monoclonal antibodies and EGFR tyrosine kinase inhibitors (TKIs) [5] (Figure 2). Initial clinical trials of these agents did not assess subjects' tumors for the absence of KRAS mutations which have since been found to confer resistance to anti-EGFR mAbs. Restricting eligibility for clinical trial participation to only patients with wild-type KRAS (wtKRAS) CRC as opposed to mutated KRAS (mutKRAS) CRC has been a crucial step in optimizing the use of EGFR targeting mAbs. Cetuximab and panitumumab are the two anti-EGFR mAbs that have demonstrated clinical benefit and have gained FDA approval for the palliative treatment of chemotherapy resistant wtKRAS metastatic colorectal cancer (mCRC). Both mAbs bind to the extracellular domain of the cell receptor and inhibit dimerization, tyrosine kinase activation, and subsequent cell signal transduction [5].

Cetuximab is a human-murine chimeric monoclonal antibody that binds to EGFR with high specificity and with a higher affinity than the natural ligands epidermal growth factor or TGF-0 [3, 12]. Thus, the mechanism of action is thought to be inhibition of ligand induced phosphorylation of EGFR [5]. Inhibition of natural ligand binding to EGFR results in several different downstream effects, all of which may contribute to the antitumor activity seen with cetuximab [4]. Cell growth and cell proliferation are turned off, apoptosis is induced, and EGFR is downregulated by internalization and degradation. Cetuximab also has been shown to decrease production of matrix metalloproteinases, enzymes which have been linked to metastatic potential [4]. Panitumumab was the first fully humanized IgG2 mAb directed against EGFR. The mechanism of action of panitumumab is similar to that of cetuximab. Panitumumab binds to EGFR, thereby preventing receptor dimerization and activation of downstream molecular signaling [9].

## 4. Cetuximab Monotherapy in Chemotherapy Refractory CRC Not Selected by KRAS Status (Table 1)

The clinical development of cetuximab predated panitumumab. Similar to many new drugs, cetuximab and panitumumab were initially evaluated as single agents in patients with mCRC resistant to all available cytotoxic chemotherapy agents. The first trial demonstrating that treatment with cetuximab alone was active in patients refractory to cytotoxic chemotherapy was reported by Saltz et al. in 2004. Patients were required to have disease resistance to irinotecan or an irinotecan containing regimen. Fifty-seven patients were treated and response rate was the only reported outcome in this phase II trial. Nine percent of subjects attained a partial response (PR) [7].

In 2007 Jonker et al. published results of a phase III trial looking at cetuximab or best supportive care (BSC) in patients refractory to fluoropyrimidine, irinotecan, and oxaliplatin. This trial is particularly notable due to the fact that it demonstrated an OS benefit in association with cetuximab. Patients were randomized to receive weekly infusions of cetuximab or BSC alone. In comparison with BSC, cetuximab treatment was associated with an improvement in OS (6.1 mos. versus 4.6 mos.: hazard ratio (HR) for death, 0.77; 95% confidence interval (CI), 0.64 to 0.92; *P* = 0.005) [8]. It is important to note that these studies were performed prior to the identification of KRAS mutations as predictors of anti-EGFR mAb resistance.

## 5. Panitumumab Monotherapy in Chemotherapy Refractory CRC Not Selected by KRAS Status (Table 1)

An open-label phase III trial of panitumumab plus BSC versus BSC alone in patients with mCRC refractory to chemotherapy demonstrated a mean PFS of 8 weeks for panitumumab and 7.3 weeks for BSC (HR 0.54; 95% CI, 0.44 to 0.66, *P* < 0.0001). Twenty-two (10%) patients in the panitumumab group attained a PR with a median response duration of 17 weeks. Importantly, crossover was allowed as part of the study design and 176 BSC patients (76%) subsequently received panitumumab which likely prevented a survival benefit to emerge [9].

## 6. Cetuximab Plus Chemotherapy for Chemoresistant mCRC Not Selected by KRAS Status (Table 1)

Chronologically, the next clinical trial efforts designed to optimize the use of anti-EGFR mAbs assessed the feasibility and benefit of concurrent chemotherapy plus mAbs. The BOND trial compared the combination of cetuximab plus irinotecan to cetuximab alone in patients with irinotecan refractory CRC. Response activity favored the combination arm, 22.9% PR versus a 10.8% PR in the cetuximab alone arm (*P* ≤ 0.001). Progression free survival also favored the combination arm: 4.1 versus 1.5 months [10]. While monotherapy did demonstrate efficacy in this trial, the better RR and PFS support the combination for this patient population [10]. This finding also suggests that cetuximab overcomes irinotecan resistance. This may occur by weakening efflux of irinotecan, impairing DNA repair activity and restoring drug-induced apoptosis.

In an open-label phase III trial (EPIC) in patients with mCRC resistant to first-line fluorinated pyrimidines and oxaliplatin, Sobrero et al. found that concurrent cetuximab plus irinotecan compared with irinotecan monotherapy was associated with both improved progression-free survival (PFS) (4.0 versus 2.6 mos; HR, 0.692; 95% CI 0.617–0.776; *P* < 0.0001) and response rate (RR) (16.4% versus 4.2%; *P* < 0.001). Overall survival (OS) however, 10.7 months versus 10 months was comparable between the two groups. The authors suggested that the lack of survival difference may have been due to the fact that 46.9% of patients in the irinotecan group ultimately were treated with cetuximab upon disease progression [11].

## 7. Development of KRAS as a Predictive Marker

RAS proteins are members of the superfamily of small GTP-binding proteins otherwise known as RAS-like GTPases. These proteins are involved in signal transduction across membranes, particularly those induced by growth factors [13]. KRAS, the human homolog of the Kirsten rat sarcoma-2 virus oncogene encodes a small GTP binding protein that acts as a self-inactivating signal transducer by cycling from GDP- to GTP-bound states in response to stimulation of cell surface receptors including EGFR [14]. KRAS can harbor oncogenic mutations that yield a constitutively active protein. Such mutations are found in approximately 30% to 50% of CRC tumors and are common in other tumor types [14]. KRAS mutations are currently the most recognized molecular predictive markers in CRC and predict the efficacy of anti-EGFR antibodies [15]. In 2006, Lièvre et al. reported that in a screening analysis for KRAS, BRAF, and PIK3CA mutations by direct sequencing tumors from 30 cetuximab treated mCRC patients wtKRAS survival compared with mutKRAS was significantly higher (16.3 versus 6.9 months; *P* = 0.016) [16].

## 8. Anti-EGFR mAB Monotherapy for Chemoresistant mCRC Selected by KRAS Status (Table 2)

Subsequently, when KRAS mutational status as a predictor of anti-EGFR mAb benefit was retrospectively assessed among 92% of 463 subjects enrolled in a prospective trial of panitumumab monotherapy versus BSC, clinical benefit was limited to wtKRAS patients. Wild-type KRAS status was associated with significantly better PFS (12.3 weeks versus 7.3 weeks) and RR (17% versus 0%). Also, in multivariate analysis wtKRAS patients had a longer OS (HR, 0.67; 95% CI, 0.55 to 0.82; treatment arms combined). The observed difference in OS between wtKRAS and mutKRAS was driven by survival benefit in wtKRAS patients who crossed over to panitumumab upon disease progression in the BSC arm [14].

These findings lead investigators to design trials that utilized KRAS mutational status as a tool to better identify groups of patients who would benefit from anti-EGFR therapy. Universal evaluation of KRAS mutational status to appropriately select mCRC patients for anti-EGFR targeted therapy also became a community oncology best practice. In 2009, the American Society for Clinical Oncology published a provisional clinical opinion in which it recommended evaluation of KRAS mutational analysis in all mCRC patients and restriction of anti-EGFR antibody therapy to wtKRAS tumors [17].

## 9. Anti-EGFR mAbs Plus Chemotherapy in Chemotherapy-Resistant mCRC Selected by KRAS Status (Table 3)

Trials evaluating chemotherapy combined with anti-EGFR mAbs in this patient population have had mixed results. The PICCOLO trial randomized patients with wtKRAS mCRC resistant to one or more prior chemotherapy regimens to treatment with irinotecan plus panitumumab or irinotecan alone. This study did not meet its primary endpoint of improved OS, but a trend was seen toward survival benefit beyond 12 months, especially in wtKRAS/wtBRAF. Patients with BRAF-mutated tumors actually had significant disbenefit with panitumumab [18]. The putative mechanism of resistance of BRAF mutations and BRAF as a clinical predictor of EGFR targeted therapy is detailed later in this paper. Sobrero et al. compared (fluorouracil, leucovorin, and irinotecan) FOLFIRI alone with FOLFIRI plus panitumumab as second-line treatment in 597 subjects. Median PFS (6.7 versus 4.9; *P* = 0.023) favored the panitumumab arm. Median OS, however, (14.5 versus 12.5, *P* = 0.366) was not improved with panitumumab, possibly because of postprogression EGFR targeted treatment received by 35% of subjects on the FOLFIRI alone control arm [19].

## 10. Anti-EGFR mAbs Monotherapy or in Combination with Chemotherapy for Therapy Naïve mCRC Selected by KRAS (Table 4)

The CRYSTAL trial compared FOLFIRI alone with FOLFIRI in combination with cetuximab as treatment of therapy naïve mCRC. This study concluded that first-line treatment with cetuximab plus FOLFIRI, as compared to FOLFIRI alone, reduced the risk of PFS only in patients with wtKRAS tumors. The hazard ratio for PFS was 0.68 (95% CI, 0.5 to 0.94) in patients with wtKRAS tumors and favored the investigational arm [20]. Unfortunately, the extended PFS was not translated to an improved OS. A smaller phase II trial, OPUS, in the same patient population compared FOLFOX alone to FOLFOX with cetuximab. There was a longer PFS (7.7 mos. versus 7.3 mos.; *P* = 0.0163) in the cetuximab arm but there was no difference in OS [21]. Again wtKRAS patients were the ones that derived benefit from the mAb treatment. In 2009, the PRIME trial confirmed that when administered as first-line therapy, panitumumab in combination with FOLFOX prolonged PFS in wtKRAS mCRC patients (9.6 mos. versus 8.0 mos.; *P* = 0.02). There was a nonsignificant increase OS observed in the investigational arm [22]. These trials therefore do not support the use of anti-EGFR mAbs as part of first-line therapy for mCRC.

## 11. Anti-EGFR mAbs Combined with anti-VEGF mAbs Selected by KRAS

Additional clinical development of anti-EGFR mAbs included investigations of these mAbs in combination with bevacizumab, an antivascular endothelial growth factor. Bevacizumab had been shown to improve RR, PFS, and OS in combination with infusional 5FU (alone or in combination with either irinotecan or oxaliplatin) as part of first- or second-line therapy of mCRC [23, 24].

## 12. Anti-EGFR mAbs with Bevacizumab in Combination with Chemotherapy for Chemotherapy-Resistant mCRC Not Selected by KRAS (Table 5)

In 2007, Saltz et al. evaluated the combination of cetuximab and bevacizumab in a phase II trial, BOND-2. Patients were naïve to both mAbs and were randomized to receive cetuximab plus bevacizumab with or without irinotecan (CBI or CB, resp.). This study concluded that the mAb doublet with or without irinotecan was feasible with toxicities similar to those expected for each agent [25]. Response rate,, time to progression (TTP), and OS favored the triple drug regimen, although these results were not confirmed in a subsequent study, BOND-2.5 [26]. This study evaluated CBI in patients with mCRC who had previously progressed on a chemotherapy plus bevacizumab regimen. The RR and TTP seen with CBI did not appear to be as encouraging as the 37% RR and 7.3 months TTP seen in the BOND-2 trial [26]. KRAS status was not evaluated in either BOND-2 or BOND-2.5.

## 13. Chemotherapy Plus Bevacizumab with or without anti-EGFR mAbs in wtKRAS Treatment Naïve mCRC (Table 6)

 Trials assessing anti-EGFR mAbs in combination with bevacizumab and chemotherapy have consistently demonstrated that double mAb regimens are inferior to bevacizumab and chemotherapy [27]. Tol et al. compared the combination of capecitabine, oxaliplatin, and bevacizumab with or without weekly cetuximab. They concluded that the addition of cetuximab to this trial resulted in a statistically significant decrease in PFS and inferior quality of life [27].

The PACCE trial in 2009 sought to combine drugs known to be effective in mCRC while avoiding overlapping toxicities. This trial evaluated panitumumab added to bevacizumab and chemotherapy as first-line treatment for mCRC [28]. The trial had four treatment arms: FOLFIRI plus bevacizumab plus or minus panitumumab and FOLFOX plus bevacizumab plus or minus panitumumab. Panitumumab was discontinued after a planned interim analysis of 812 FOLFOX patients demonstrated worse PFS and OS in the panitumumab arm [28]. The negative outcome of this study raised the possibility of negative interactions between EGFR inhibitors and bevacizumab when combined with chemotherapy. Notably, subjects with mutKRAS disease had a trend towards poorer survival rates when treated with panitumumab [28]. A report from a phase III study investigating capecitabine, oxaliplatin, and bevacizumab with or without cetuximab in first-line mCRC (CAIRO2) also showed inferior PFS in the investigational arm (10.7 versus 9.8 mos.; *P* = 0.019) [30].

Saltz and colleagues compared FOLF (leucovorin 400 mg/m^2^ plus 5-FU 400 mg/m^2^ bolus followed by 2.4 gram 48-hour infusion every 48 hours) in combination with bevacizumab and cetuximab to FOLFOX plus bevacizumab for first-line treatment of mCRC. The authors hoped that replacing oxaliplatin with two targeted therapies would achieve superior 12-month PFS with a better tolerated regimen. The trial demonstrated that the combination of FOLF + bevacizumab + cetuximab is inferior to FOLFOX plus bevacizumab in terms of PFS at 12 months (44.6% versus 32.3%; *P* = 0.03). Overall survival also was not statistically different (21.3 mos. versus 19.5 mos., *P* = 0.13) although the trial was not powered for OS. Furthermore, patient satisfaction was lower in the investigational arm possibly because cetuximab specific side effects are more troubling to patients than oxaliplatin specific side effects [29]. This trial adds to evidence from the PACCE and Tol trials that combining anti-EGFR mAbs to bevacizumab based chemotherapy is an inappropriate treatment for mCRC.

## 14. Recently Published Trials of Anti-EGFR mAbs Combined with Other Targeted Therapies (Table 7)

The addition of brivanib alaninate, a TKI targeting vascular endothelial and fibroblast growth factor receptors, to cetuximab has shown encouraging activity in an early phase clinical trial. However, despite positive effects on PFS and objective response rates, the combination of these two drugs did not significantly improve OS [31]. The addition of dalotuzumab, an anti-insulin-like growth factor receptor (IGFR) mAb to cetuximab and irinotecan worsened PFS and OS in pts with chemorefractory wtKRAS mCRC in a phase II/III study conducted by Watkins et al. [32].

## 15. Cetuximab in the Adjuvant Setting (Table 8)

Typical of drug development in oncology, once a drug demonstrates clinical benefit in metastatic disease, its efficacy is often assessed as adjuvant therapy for earlier stage, operable tumors. Intergroup study N0147 evaluated the addition of cetuximab to adjuvant FOLFOX-6 in patients who had undergone complete resection of stage III colon cancer. Worsened disease-free survival (DFS) and a trend toward inferior OS was observed in patients with mutKRAS treated with cetuximab [33]. In 2010, the same intergroup study reported updated data on 156 subjects treated on arms with FOLFIRI alone or with cetuximab. FOLFIRI resulted in a 3-year DFS lower than that expected for FOLFOX. However, trends for improved 3-year DFS (80% versus 65%; HR = 0.6 (95% CI, 0.3 to 1.1); *P* = 0.09) and 3-year OS (90% versus 83%; HR = 0.4 (95% CI, 0.1 to 1.0); *P* = 0.04) with the addition of cetuximab to FOLFIRI were observed in patients with resected stage III CRC regardless of KRAS status [34]. While only 27 subjects had wtKRAS tumors treated with adjuvant FOLFIRI plus cetuximab, the resulting 3 year DFS of 88% is provocative. These outcomes from N0147 raise two important hypotheses: (1) the possibility that chemotherapy choice may be an important factor in the further development of anti-EGFR mAbs as adjuvant therapy and (2) KRAS mutational status may be an important factor in personalizing adjuvant treatment for resected stage III and possibly high risk-stage II CRC.

PETACC-8 a randomized, multicenter, European phase III trial is comparing the efficacy of cetuximab plus FOLFOX-4 with that of FOLFOX-4 alone in patients with stage III colon cancer. The primary end point of this study is DFS time analyzed after a minimum followup of 3 years per patient [35].

## 16. Monoclonal Antibodies as Neoadjuvant Therapy for Potentially Resectable Stage IVA mCRC

The role of neoadjuvant chemotherapy for unresectable colorectal liver metastasis has been established as a method of downsizing tumors for the purpose of curative resection. Folprecht et al. conducted an open-label study randomizing patients to receive neoadjuvant cetuximab plus either FOLFOX6 or FOLFIRI (CELIM trial). The primary endpoint was RR. There was no difference in the two groups in terms of response rates (difference 11%, 95% CI −8–30; odds ratio (OR) 1.62, 0.74–3.59; *P* = 0.23). Not surprisingly a retrospective analysis based on KRAS status showed that the response in the wtKRAS tumors was 70% versus 41% in the mutKRAS tumors (OR 3.42, 95% CI 1.35–8.66; *P* = 0.008). Furthermore, tumors that were both wtKRAS and wtBRAF had a 72% RR as compared to the tumors that harbored a mutation in either gene (72% versus 40%, *P* = 0.003). The matured survival data recently reported by this group showed no difference in OS between the wtKRAS and mutKRAS groups (36.1 versus 27.4 months) however [36].

## 17. Monoclonal Antibodies and Rectal Cancer

The German Rectal Cancer Study Group reported that preoperative chemoradiation improves local control and sphincter preservation and is associated with reduced toxicity but does not improve survival compared with postoperative therapy [37]. Retrospective analyses have demonstrated lower pathologic complete response (pCR) rates and shorter DFS in patients with rectal cancer expressing EGFR who were treated with neoadjuvant radiation therapy. This suggested that radiosensitivity might be increased by targeting the EGFR [38]. Studies are investigating the role of radiation sensitizing agents and the combination of radiotherapy with targeted agents in an attempt to improve local control and DFS [37]. Phase I/II studies of combinations of cetuximab and chemoradiotherapy have demonstrated that cetuximab can be given safely, but the pCR rates have been low [38]. To date, these studies have failed to demonstrate correlation between KRAS status and efficacy of mAbs.

## 18. Emerging Strategies to Identify and Overcome Anti-EGFR Resistance

While only KRAS mutations are currently validated as predictive markers to treatment with mAbs, many wtKRAS patients still do not benefit from these drugs. Research is now concentrated on other EGFR downstream effectors, proteins that may be potential predictors of response to mAbs. BRAF proteins are downstream from KRAS and studies have shown that BRAF mutations also play a role in resistance to mAbs. The V600E is the most common point mutation that involves the BRAF gene and is present in approximately 10% of CRCs [5]. Studies looking at the response to mAbs in wtBRAF tumors have shown that tumors that are both wtKRAS and wtBRAF have better response rates, suggesting BRAF as a therapeutic target. This was demonstrated in the DUX study in which wtKRAS tumors had a 41% RR, but when wtKRAS/wtBRAF tumors were analyzed the RR was 52% [39]. A retrospective analysis of mCRC patients who received therapy with EGFR mAbs showed that 11 out of 113 (10%) tumors had a BRAF V600E mutation, and none of them responded to EGFR monotherapy [40].

Two studies retrospectively evaluated simultaneous mutations in KRAS and BRAF and response to mAbs. The Laurent-Puig group collected samples from 173 patients with mCRC. One hundred and sixteen of these tumors were wtKRAS, 100 of which were also wtBRAF. They found that BRAF mutations were weakly associated with lack of response (*P* = 0.063) but strongly associated with shorter PFS (*P* < 0.001) and shorter OS (*P* < 0.001) [41]. Similarly, Ruzzo et al. found that among 66 wtKRAS tumors, nine or 14% were mutBRAF. Wild-type BRAF tumors had improved RR (33%; *P* = 0.04), and although there was a trend towards prolonged PFS (5.1 versus 3.3 mos.; *P* = 0.076), this was not statistically significant [42].

Bokemeyer et al. analyzed the pooled data of 845 subjects with wtKRAS tumors from the Crystal and the Opus trials. BRAF mutations were present in 70 of the 800 evaluable tumors. They found that the prognosis was poorer in the mutBRAF tumors as compared to the wtBRAF tumors. The small number of mutBRAF tumors in these studies reflects the frequency of this mutation [43]. Although data to this point does not merit using BRAF mutational status to guide anti-EGFR mAbs selection, further investigation is warranted.

One of the main pathways activated by EGFR is the P13 K/PTEN/AKT signaling cascade. Mutations in the PIK3CA gene, which encodes for the p110 catalytic subunit of the PI3 K, occur in about 15% of tumors [5]. These mutations can be found together with KRAS and BRAF mutations, and this makes it difficult to define the relative contribution of this mutation to anti-EGFR mAbs resistance. Apart from KRAS and BRAF, two other groups of molecules related to the EGFR pathway have also emerged as potential biomarker candidates: EGFR ligands and fragment crystallizable-gamma receptors (Fc*γ*R) polymorphisms. EGFR is activated by a variety of ligands such as amphiregulin (AREG), epiregulin (EREG), and transforming growth factor-*α*. It has been shown that higher levels of AREG and EREG have a positive predictive value in determining response to cetuximab, and when treated with cetuximab these tumors have better PFS [40].

Although attempts at developing drugs targeting KRAS have largely been unsuccessful, several BRAF inhibitors have been discovered. Sorafenib is an oral multikinase inhibitor that targets both wtBRAF and oncogenic BRAF V600E and has in vitro activity in CRC cell lines with this mutation. The NEXIRI phase II trial combining sorafenib with irinotecan showed that this combination in patients with chemotherapy-resistant mutKRAS tumors has encouraging activity. Median PFS and OS were 3.5 months and 7.7 months, respectively [45]. PLX4032 (RG7204) is an oral inhibitor of the mutBRAF kinase with pronounced activity in mutBRAF melanoma patients. A phase I study of PLX4032 included a cohort of patients with mutBRAF mCRC. Clinical activity in these patients was modest (PFS 3.7 months) but does support mutant BRAF as a therapeutic target in colorectal cancers [46].

The issue of cross-resistance between panitumumab and cetuximab was evaluated in a trial of twenty patients with wtKRAS mCRC treated with cetuximab and irinotecan followed by panitumumab monotherapy after progression. No patients responded. The PFS was 1.7 months and the OS was 5.2 months. At this time, the use of panitumumab following cetuximab failure cannot be recommended [47].

## 19. The Monoclonal Antibody Rash (Table 9)

The most common side effects of anti-EGFR mAbs are dermatologic including an acne-like rash, xerosis (dry skin), and fissures of the skin. Although some degree of acneiform rash occurs in most patients, severe eruptions resulting in significant pain or infectious sequelae are less common [48]. Available retrospective evidence suggests that the appearance and severity of a skin rash is positively correlated with objective tumor response to mAb therapy and with OS in mCRC [49]. In patients receiving cetuximab or panitumumab monotherapy for mCRC, longer survival times were observed in patients with rash of any grade as compared with patients experiencing no rash [48]. Retrospectively observed rash-to-survival correlations suggest that individualized dose titration based on the appearance and severity of skin rash may allow for optimization of therapy and has led to the initiation of “dose-to-rash” trials [49]. In the EVEREST trial, patients with no or slight skin reactions after 22 days of standard cetuximab therapy were randomized to continue receiving a standard dose or to begin a dose escalation every two weeks until a grade 2 skin toxicity developed or a ceiling dose of 500 mg/m^2^ was achieved. As compared with the standard-dose arm, the dose-escalation arm demonstrated an improved RR (risk ratio: 30% versus 13%). This finding supports the relationship between tumor RR, mAb dose, and skin rash [50]. The NCIC Clinical Trials CO.17 trial demonstrated a strong correlation between benefit from cetuximab therapy and both rash and KRAS status. Rash was graded weekly by NCI CTC 2.0 criteria. More severe rash was observed in the subjects with wtKRAS tumors than in the mutKRAS patients (57% versus 44.4%; *P* = 0.08). In addition, rash severity was positively correlated with PFS and OS in the wtKRAS patients [44].

STEPP was a phase II open-label trial of skin toxicity evaluation in mCRC patients receiving panitumumab plus FOLFIRI or irinotecan only in the chemoresistant setting. This randomized study examined differences between preemptive and reactive skin treatments for the rash associated with panitumumab therapy. Patients randomized to the preemptive treatment arm received daily skin treatment and oral doxycycline from 24 hours before their first dose of panitumumab through week 6. Patients in the reactive treatment arm received treatment only after development of skin toxicity. As compared with the reactive treatment, preemptive treatment reduced the incidence of grade 2 or greater skin toxicities by more than 50% without additional side effects [51].

## 20. Electrolyte Imbalances

Grade 3/4 hypomagnesemia has been consistently reported across clinical trials of cetuximab and panitumumab [49]. Some data point to a direct relationship between the duration of cetuximab exposure and hypomagnesemia. The mechanisms responsible for hypomagnesemia in association with anti-EGFR mAbs have not been well defined [52]. Increased EGFR expression in the ascending loop of Henle, where 70% of filtered magnesium is reabsorbed, may result in damage to the renal tubule, and interfere with magnesium transport, causing a magnesium wasting syndrome [52, 53]. Symptoms of hypomagnesemia can be cardiovascular, neuromuscular, or behavioral. Hypocalcemia has been reported in association with hypomagnesemia and can contribute to neuromuscular symptoms. This hypomagnesemic hypocalcemia can only be corrected by replacing magnesium levels. The pathophysiology of hypocalcemia in this setting is related to hypomagnesemia-induced PTH resistance [54].

Hypokalemia has similarly been associated with anti-EGFR therapy, although to a lesser extent than hypomagnesemia. Patients with grade I hypomagnesemia are generally asymptomatic and do not require replacement therapy. In patients with grade 2 hypomagnesemia, oral supplementation is generally ineffective and poorly tolerated due to diarrhea. Weekly intravenous treatment with magnesium sulfate 4 g has been shown to be effective for patients with magnesium levels of 0.9 to 1.0 mg/dL (0.37–0.41 mmol/L). For patients with grade 2 hypomagnesemia who are asymptomatic and do not have cardiac risk factors, weekly monitoring without magnesium supplementation may be considered. Grades 3-4 hypomagnesemia are associated with symptoms of fatigue, cramps and somnolence which can mistakenly be attributed to cytotoxic chemotherapy and therefore go unreported. Replacement therapy is particularly important for these patients to prevent cardiac arrhythmias and sudden cardiac death [54].

## 21. Conclusion

Although anti-EGFR targeted therapy has become an important component of the treatment of mCRC, only a subset of patients benefit from these treatments. It is becoming evident that KRAS mutational status is only a small aspect of why some patients respond to these treatments while others do not. There are many trials underway investigating the role of anti-EGFR antibodies either as single agents or in combination with other targeted therapies (Table 10). As summarized in this paper, clinical and translational investigators have been productive in trying to identify either optimal strategies or expanded roles for EGFR-targeted therapy as treatment for colorectal cancer. Unfortunately, despite these efforts, data to date suggests that anti-EGFR mAb use should be restricted to patients with wtKRAS mCRC resistant to fluorinated pyrimidines, oxaliplatin, and irinotecan (or for patients intolerant to irinotecan) either as monotherapy or in combination with irinotecan.

## Figures and Tables

**Table 1:** Trials using Anti-EGFR mABs for chemotherapy-resistant mCRC not selected by KRAS status.

Trial (author)	Phase	Protocol	Number enrolled	Results		HR (95% CI)	*P* value
				PFS	OS		
— (Saltz et al.) [7]	II	Cetuximab	57	PR 9%			

CO.17 (Jonker et al.) [8]	III	Cetuximab BSC	287 285		6.1 mos. 4.6 mos.	OS: 0.77 (0.64–0.92)	<0.005

— (Peeters et al.) [9]	III	Panitumumab + BSC BSC	231 232	8 wks. 7.3 wks.		PFS: 0.54 (0.44–0.66)	<0.0001

BOND (Cunningham et al.) [10]	II	Cetuximab + irinotecan	218	PR 22.9%		PR: N/A	<0.001
		cetuximab	211	PR 10.8%			

EPIC (Sobrero et al.) [11]	III	Cetuximab + Irinotecan	648	4.0 mos.	10.7 mos.	PFS: 0.69 (0.61–0.77) OS: 0.97 (0.84–1.11) RR: N/A	<0.0001 0.71 <0.0001
				RR 16.4%		
		Irinotecan	650	2.6 mos.	10.0 mos.	
				RR 4.2%		

BSC: best supportive care, PR: partial response, OS: overall survival, PFS: progression-free survival, RR: response rate, and N/A: not applicable.

**Table 2:** Trials using Anti-EGFR mABs as monotherapy for chemoresistant mCRC selected by KRAS status.

Trial (author)	Phase	KRAS	Protocol	Number enrolled	Results		HR (95% CI)	*P* value
					PFS	OS		
— (Amado et al.) [14]	III	WT	Panitumumab BSC	124 119	12.3 wks. 7.3 wks.	8.1 mos. 7.6 mos.	PFS: 0.45 (0.34–0.59) OS: 0.99 (0.75–1.29)	<0.001 NR
		MUT	Panitumumab BSC	84 100	7.4 wks. 7.3 wks.	4.9 mos. 4.4 mos.	PFS: 0.99 (0.73–1.36) OS: 1.02 (0.75–1.39)	NR NR

BSC: best supportive care, WT: wild type, MUT: mutated, and NR: not reported.

**Table 3:** Trials using Anti-EGFR mABs with chemotherapy in chemoresistant mCRC selected by KRAS status.

Trial (author)	Phase	KRAS	Protocol	Number enrolled	Results		HR (95% CI)	*P* value
					PFS	OS		
PICCOLO (Seymour et al.) [18]	III	WT WT	Irinotecan + panitumumab Irinotecan	Total number of patients = 324		10.4 mos. 10.5 mos.	OS: 0.91 (0.73–1.14)	0.44

Study 181 (Sobrero et al.) [19]	III	WT	FOFIRI + panitumumab FOLFIRI	303 294	6.7 mos. 4.9 mos.	14.5 mos. 12.5 mos.	PFS: 0.82 (0.69–0.97) OS: 0.92 (0.78–1.10)	0.023 0.366
		MUT	FOFIRI + panitumumab FOLFIRI	238 248	5.3 mos. 5.4 mos.	11.8 mos. 11.1 mos.	PFS: 0.95 (0.78–1.14) OS: 0.93 (0.77–1.13)	0.561 0.482

WT: wild type, MUT: mutated, PFS: progression free survival, and OS: overall survival.

**Table 4:** Anti-EGFR mAbs monotherapy or in combination with chemotherapy for therapy naïve mCRC selected by KRAS.

Trial (author)	Phase	KRAS	Protocol	Number enrolled	Results		HR (95% CI)	*P* value
					PFS	OS		
Crystal (Van Cutsem et al.) [20]	III	WT	FOLFIRI + cetuximab FOLFIRI	172 176	9.9 mos. 8.7 mos.	24.9 mos. 21.0 mos.	PFS: 0.68 (0.50–0.94) OS: 0.84 (0.64–1.11)	0.07 NR
		MUT	FOLFIRI + cetuximab FOLFIRI	105 87	7.6 mos. 8.1 mos.	17.5 mos. 17.7 mos.	PFS: 1.07 (0.71–1.61) OS: 1.03 (0.74–1.44)	0.44 NR

OPUS (Bokemeyer et al.) [21]	II	WT	FOLFOX + cetuximab FOLFOX	61 73	7.7 mos. 7.2 mos.		PFS: 0.57 (0.35–0.90)	0.0163
		MUT	FOLFOX + cetuximab FOLFOX	52 47	5.5 mos. 8.6 mos.		PFS: 1.83 (1.09–3.05)	0.192

PRIME (Douillard et al.) [22]	III	WT	FOLFOX + panitumumab FOLFOX	325 331	9.6 mos. 8.0 mos.	23.9 mos. 19.7 mos.	PFS: 0.80 (0.66–0.97) OS: 0.83 (0.67–1.01)	0.02 0.072
		MUT	FOLFOX + panitumumab FOLFOX	221 219	7.3 mos. 8.8 mos.	15.5 mos. 19.3 mos.	PFS: 1.29 (1.04–1.62) OS: 1.24 (0.98–1.57)	0.02 0.68

WT: wild type, MUT: mutated, PFS: progression free survival, OS: overall survival, and NR: not reported.

**Table 5:** Trials using anti-EGFR mAbs plus bevacizumab in chemotherapy-resistant mCRC not selected by KRAS.

Trial (author)	Phase	Protocol	Number enrolled	Results	HR (95% CI)	*P* value
BOND-2 (Saltz et al.) [25]	II	Cetuximab + bevacizumab	40	TTP 4.9 mos.		
				RR 20%		
				OS 11.4 mos.		
		Cetuximab + bevacizumab + irinotecan	43	TTP 7.3 mos.		
				RR 20%		
				OS 11.4 mos.		

BOND-2.5 (Segal et al.) [26]	II	Cetuximab + bevacizumab + irinotecan	33
				TTP 3.9 mos.		
				9% PR MS 10.6 mos.		

TTP: time to progression, RR: response rate, OS: overall survival, PR: partial response, and MS: median survival.

**Table 6:** Trials using anti-EGFR mAbs plus bevacizumab in chemotherapy naïve mCRC selected by KRAS.

Trial (author)	Phase	KRAS	Protocol	Number enrolled	Results		HR (95% CI)	*P* value
					PFS	OS		
— (Tol et al.) [27]	III	WT	Capecitabine + oxaliplatin + bevacizumab + cetuximab	158	10.5 mos.	21.8 mos.	PFS: NR	0.3
			Capecitabine + oxaliplatin + bevacizumab	156	10.6 mos.	22.4 mos.	OS: NR	0.64
		MUT	Capecitabine + oxaliplatin + bevacizumab + cetuximab	98	8.1 mos.	17.2 mos.	PFS: NR	0.003
			Capecitabine + oxaliplatin + bevacizumab	108	12.5 mos.	24.9 mos.	OS: NR	0.03

PACCE (Hecht et al.) [28]	III	WT	FOLFOX + panitumumab + bevacizumab	201	9.8 mos.	20.7 mos.	PFS: 1.36 (1.04–1.77)	NR
			FOLFOX + bevacizumab	203	11.5 mos.	24.5 mos.	OS: 1.89 (1.30–2.75)	0.045
		MUT	FOLFOX + panitumumab + bevacizumab	135	10.4 mos.	19.3 mos.	PFS: 1.25 (0.91–1.71)	NR
			FOLFOX + bevacizumab	125	11.0 mos.	19.3 mos.	OS: 1.02 (0.67–1.54)	NR

— (Saltz et al.) [29]	III	WT	mFOLFOX + bevacizumab	49	10.9 mos.	18.8 mos.	PFS: NR	0.99
			FOLF + cetuximab + bevacizumab	46	8.8 mos.	21.5 mos.	OS: NR	0.96
		MUT	mFOLFOX + bevacizumab	36	12.1 mos.	22.4 mos.	PFS: NR	<0.01
			FOLF + cetuximab + bevacizumab	33	8.0 mos.	20.3 mos.	OS: NR	0.11

WT: wild type, MUT: mutated, PFS: progression free survival, OS: overall survival, and NR: not reported.

**Table 7:** Trials using anti-EGFR MAbs with other targeted therapies in chemotherapy-resistant mCRC in KRAS WT patients.

Trial (author)	Phase	Protocol	Number enrolled	Results		HR (95% CI)	*P* value
				PFS	OS		
AGITG CO.20 (Siu et al.) [31]	III	Cetuximab + brivanib alaninate cetuximab	376 374	5.0 mos. 3.4 mos.	8.8 mos. 8.1 mos.	PFS: 0.72 (0.62–0.84) OS: 0.88 (0.74–1.03)	<0.0001 0.12

— (Watkins et al.) [32]	II/III	Dalotuzumab 10 mg/kg 1 week + cetuximab + irinotecan versus	Total enrolled 345 WT	3.3 mos.	10.8 mos.	PFS: NR OS: NR	
		dalotuzumab 7.5 mg/kg q 2 weeks versus		5.4 mos.	11.6 mos.		
		placebo + cetuximab + irinotecan		5.6 mos.	14.0 mos.		

WT: wild type, PFS: progression free survival, OS: overall survival, NR: not reported.

**Table 8:** Clinical trials of cetuximab in stage III colon cancer.

Trial (first author)	Phase	Protocol	Number enrolled	Results	HR (95% CI)	*P* value
NCCTG NO147 (Goldberg et al.) [33]	III	FOLFOX 6 + cetuximab	318	3-year DFS 62.3% OS 79.1%	DFS: 1.48 (1.08–2.03) OS: 1.67 (1.00–2.80)	0.02 0.07
		FOLFOX 6	340	3-year DFS 70.3% OS 86.1%		

NCCTG NO17 (Huang et al.) [34]	III	FOLFIRI + cetuximab	45	3-year DFS 80% OS 90%	DFS: 0.6 (0.3–1.1) OS: 0.4 (0.1–1.0)	0.09 0.04
		FOLFIRI	111	3-year DFS 65% OS 83%		

DFS: disease-free survival, OS: overall survival.

**Table 9:** The relationship between the development of rash and clinical outcome when using anti-EGFR mAbs in mCRC.

Trial (first author)	KRAS	Protocol	Grade of rash	Results		HR (95% CI)	*P* value
				PFS	OS		
— (O'Callaghan et al.) [44]	WT	BSC versus	0/1	1.9 mos.	5 mos.	PFS: 0.57 (0.38–0.86) OS: 0.85 (0.56–1.31)	0.008 0.46
		cetuximab		2.2 mos.	8 mos.		
	WT	BSC versus	2+	1.9 mos.	5 mos.	PFS: 0.32 (0.21–0.49) OS: 0.52 (0.34–0.8)	<0.001 0.003
		cetuximab		5.1 mos.	9.8 mos.		
	MUT	BSC versus	0/1	1.9 mos.	5 mos.	PFS: 0.97 (0.65–1.46) OS: 1.47 (0.95–2.27)	0.89 0.08
		cetuximab		1.8 mos.	4.0 mos.		
	MUT	BSC versus	2+	1.9 mos.	5 mos.	PFS: 0.82 (0.52–1.3) OS: 0.82 (0.47–1.41)	0.89 0.46
		cetuximab		1.8 mos.	6.6 mos.		

WT: wild type, MUT: mutated, PFS: progression-free survival, and OS: overall survival.

**Table 10:** 

Title	Condition	Biological intervention	Drug combo	1st line versus 2nd line versus adjuvant	Identifier	Comments
A study with neoadjuvant mFOLFOX7 plus cetuximab to determine the surgical conversion rate for unresectable colorectal cancer with metastasis confided to the liver (2008)	Metastatic colorectal cancer	Cetuximab	FOLFOX	Neoadjuvant	NCT00803647	Surgical candidates only

Phase 2A study of NPC-1C chimeric monoclonal antibody to treat pancreatic and colorectal cancer (2009)*	Metastatic colorectal cancer; metastatic pancreatic cancer	NPC-1C (ensituximab)		2nd line	NCT01040000	Refractory to standard treatment

EMD 5257 in combination with cetuximab and irinotecan in K-RAS Wild-type metastatic colorectal cancer (2009)**	Metastatic colorectal cancer	EMD525797 and cetuximab	irinotecan	2nd line	NCT008475	Randomized, no placebo

Dual Epidermal growth factor receptor inhibition with erlotinib and panitumumab with or without chemotherapy for advanced colorectal cancer (2009)	Colorectal cancer	Panitumumab + erlotinib	irinotecan	2nd line	NCT00940316	Refractory to FOLFOX

Irinotecan hydrochloride and cetuximab with or without ramucirumab in treating patients with advanced colorectal cancer with progressive disease after treatment with bevacizumab-containing chemotherapy (2010)	Colorectal cancer	Cetuximab and ramucirumab	irinotecan	2nd line	NCT01079780	Refractory to bevacizumab containing chemotherapy

A study of IMC-1121b or IMC-18f1 in colorectal cancer (2010)	Colon cancer Rectal cancer	IMC-1121b + IMC-18F1	mFOLFOX-6	2nd line	NCT01111604	Refractory to irinotecan-based first-line chemotherapy

A study of perioperative chemotherapy plus panitumumab in patients with colorectal cancer liver metastases (2010)	Colorectal cancer; liver metastasis	Panitumumab	oxaliplatin, 5-FU	Neoadjuvant	NCT01260415	Surgical candidates only

FOLFOXIRI plus panitumumab patients with metastatic KRAS wild-type colorectal cancer with liver metastases only (2010)	Colorectal cancer	Panitumumab	FOLFOXIRI	1st line	NCT01226719	Nonrandomized, no placebo

Study of first-line single-agent panitumumab in frail elderly patients with advanced wild-type K-RAS colorectal cancer (FRAIL) (2010)	Colorectal cancer	Panitumumab		1st line	NCT01126112	Nonrandomized, no placebo

Study of panitumumab-capecitabine oxaliplatin in wild-type K-RAS colorectal cancer patients (2010)	Metastatic colorectal cancer	Panitumumab	Capecitabine/oxaliplatin	1st line	NCT01215539	Nonrandomized, no placebo

Study assessing potential predictive tumor markers in metastatic colorectal cancer (PULSE) (2010)	Metastatic colorectal cancer	Panitumumab	FOLFOX	2nd line	NCT01288339	Wild-type K-RAS according to IGFRp and MMP-7 expression

Panitumumab and bortezomib for patients with advanced colorectal cancer (2011)	Colorectal cancer	Panitumumab + bortezomib		2nd line	NCT01504477	Refractory to standard treatment

Efficacy and safety of GS-6624 with FOLFIRI as second-line treatment in colorectal adenocarcinoma (2011)	Colorectal cancer	GS-6624	FOLFIRI	2nd line	NCT01479465	Randomized, placebo-controlled

Neoadjuvant radiochemotherapy combined with panitumumab in locally advanced KRAS wild-type rectal cancer (NEOREC-1) (2011)	Rectal cancer	Panitumumab		Neoadjuvant	NCT01443377	Surgical candidates only

Safety study of the combination of panitumumab, irinotecan, and everolimus in the treatment of advanced colorectal cancer (PIE) (2011)	Colorectal cancer	Panitumumab	Everolimus, irinotecan	2nd line	NCT01139138	FOLFOX refractory

FOLFOXIRI plus panitumumab in K-RAS and BRAF wild-type metastatic colorectal cancer (TRIP) (2011)	Metastatic colorectal cancer	Panitumumab	FOLFOXIRI	1st line	NCT01358812	K-RAS and BRAF wild-type; nonrandomized, no placebo

FOLFOXIRI with or without panitumumab in metastatic colorectal cancer (VOLFI) (2011)	Metastatic colorectal cancer	Panitumumab	FOLFOXIRI	2nd line	NCT01328171	Randomized, no placebo

*NPC-1 is a chimeric immunoglobulin molecule thought to have specific immunoreactivity with colon and pancreas cancer.

**EMD 525797 humanized monoclonal antibody directed against the human alpha v integrin subunit with potential antiangiogenic and antineoplastic activities including inhibition of endothelial cell-cell interactions, endothelial cell-matrix interactions, and integrin-mediated tumor angiogenesis and metastasis in alphavbeta3-expressing tumor cells.
